# Are *KRAS/BRAF* Mutations Potent Prognostic and/or Predictive Biomarkers in Colorectal Cancers?

**DOI:** 10.2174/187152012799014968

**Published:** 2012-02

**Authors:** Tomoya Yokota

**Affiliations:** Division of Gastrointestinal Oncology, Shizuoka Cancer Center, Shizuoka, Japan

**Keywords:** Anti-EGFR monoclonal antibody, *BRAF*, Cetuximab, Chemotherapy, Clinicopathological features, Colorectal cancer, Driver mutation, EGFR, *KRAS* codon 12, *KRAS* codon 13, *KRAS* p.G13D, Panitumumab, Predictive marker, Prognostic biomarker, Screening.

## Abstract

*KRAS *and *BRAF *mutations lead to the constitutive activation of EGFR signaling through the oncogenic Ras/Raf/Mek/Erk pathway. Currently, *KRAS *is the only potential biomarker for predicting the efficacy of anti-EGFR monoclonal antibodies (mAb) in colorectal cancer (CRC). However, a recent report suggested that the use of cetuximab was associated with survival benefit among patients with p.G13D-mutated tumors. Furthermore, although the presence of mutated *BRAF *is one of the most powerful prognostic factors for advanced and recurrent CRC, it remains unknown whether patients with *BRAF*-mutated tumors experience a survival benefit from treatment with anti-EGFR mAb. Thus, the prognostic or predictive relevance of the *KRAS *and *BRAF *genotype in CRC remains controversial despite several investigations. Routine *KRAS*/*BRAF *screening of pathological specimens is required to promote the appropriate clinical use of anti-EGFR mAb and to determine malignant phenotypes in CRC. The significance of *KRAS*/*BRAF *mutations as predictive or prognostic biomarkers should be taken into consideration when selecting a *KRAS*/*BRAF *screening assay. This article will review the spectrum of *KRAS*/*BRAF *genotype and the impact of *KRAS*/*BRAF *mutations on the clinicopathological features and prognosis of patients with CRC, particularly when differentiating between the mutations at *KRAS *codons 12 and 13. Furthermore, the predictive role of *KRAS*/*BRAF *mutations in treatments with anti-EGFR mAb will be verified, focusing on *KRAS *p.G13D and *BRAF *mutations.

## INTRODUCTION

The development of colorectal cancer (CRC) is a multistep process that occurs because of the accumulation of several genetic alterations, including chromosomal abnormalities, gene mutations, and epigenetic modifications involving several genes that regulate proliferation, differentiation, apoptosis, and angiogenesis [1, 2].

Of the various genetic alterations, an important molecular target for metastatic CRC treatment is the epidermal growth factor receptor (EGFR). EGFR, also known as HER1 or ErbB, is a 170-kD receptor tyrosine kinase and belongs to the ErbB receptor family. There are four members in the ErbB receptor family: ErbB1 (EGFR, HER1), ErbB2 (HER2/neu), ErbB3 (HER3), and ErbB4 (HER4). The binding of several specific ligands, such as EGF, TGF-α, or amphiregulin, results in the dimerization of EGFR and subsequent phosphorylation of several tyrosine residues [3, 4]. These phosphorylated tyrosines serve as binding sites for several signal transducers that initiate multiple signaling pathways, including the Ras/Raf/MAP/MEK/ERK and/or PTEN/PI3K/Akt pathways. Although EGFR plays important roles in cell differentiation and proliferation in normal cells, the activation of EGFR signaling is frequently observed in CRC cells, where it results in cell proliferation, migration and metastasis, evasion of apoptosis, or angiogenesis [5]. Approximately 35% CRC tissues carry a mutation at codon 12 or 13 of *KRAS* that leads to the constitutive activation of EGFR downstream pathways [6-10].

Information on the *KRAS*/*BRAF* genotype is also extremely useful when selecting systemic chemotherapy for advanced and recurrent patients with CRC, where it can help identify patients with poor prognoses. *KRAS* and *BRAF* are currently under focus as potential prognostic and predictive biomarkers in patients with metastatic diseases treated with anti-EGFR monoclonal antibodies (mAb), such as cetuximab and panitumumab [11-14]. Several retrospective analyses revealed that cetuximab treatment is ineffective in patients with *KRAS* mutations, thereby suggesting that the *KRAS*genotype is a useful predictive biomarker for cetuximab or panitumumab therapy in CRC [11-13, 15]. It has also been suggested that wild-type *BRAF* is required for a successful response to panitumumab or cetuximab therapies in patients with metastatic CRC [9, 10, 16, 17]. However, the prognostic relevance of the *KRAS* genotype in CRC remains controversial despite several multi-institutional investigations since the 1990s [18-22].

In this article, I will review the spectrum of the *KRAS*/*BRAF* genotype and the clinical outcomes of *KRAS*/*BRAF* mutations in patients with CRC. The prognostic and/or predictive impact of *KRAS*/*BRAF* mutations will then be discussed, focusing on the difference between mutations at *KRAS* codons 12 and 13.

## POTENTIAL PREDICTIVE BIOMARKERS FOR ANTI-EGFR THERAPY

The molecular mechanisms underlying response or resistance to anti-EGFR mAb still remain largely unknown. However, the clinical predictive factors that indicate the response or resistance to anti-EGFR therapy should be identified before beginning such a treatment in patients with CRC to prevent drug-induced toxicity and avoid unnecessary expenses. The main research areas in this setting have been focusing on the role of (i) EGFR protein expression, (ii) EGFR gene copy number, (iii) EGFR gene mutations, (iv) overexpression of EGFR ligands (such as epiregulin and amphiregulin), (v) methylation of the *EGFR* promoter, and (vi) markers of EGFR downstream signaling [8, 9, 23-28]. 

For initial clinical trials, patients with metastatic CRC were selected if they had tumors positive for the expression of EGFR as detected by immunohistochemistry (IHC). However, cetuximab is also effective in patients with CRC having tumors that do not express EGFR when examined by IHC [29]. Indeed, EGFR is overexpressed in 30%–85% patients with CRC. Therefore, the level of EGFR protein expression has proved to be poorly associated with sensitivity to anti-EGFR mAb. Inconsistent methodology and interpretation of EGFR IHC expression in tumor samples may be an explanation for this. Inter-observer variability in the definitions of the expression EGFR may depend on the tissue fixation technique used, possibly leading to false negative samples by IHC using paraffin-embedded tumor tissues. Significant differences in EGFR IHC expression between a patient’s primary tumor and their metastatic tissue specimen may be another explanation. The primary tumor is frequently used to establish the patient’s EGFR status, but metastases are treated with cetuximab. A third explanation is that high-affinity EGFRs are the predominant biologically active receptors that lead to the activation of protein tyrosine kinase, thereby contributing significantly to signal transduction [30]. However, the anti-EGFR antibodies that are most commonly used do not distinguish between high-affinity and low-affinity EGFRs [31]. Another potential explanation may be the potential of cetuximab to induce antibody-dependent cell-mediated cytotoxicity (ADCC) despite an equivalent pharmacological EGFR blockade.

In a small fraction of CRCs, the overexpression of EGFR is frequently associated with amplification of the gene. The EGFR gene copy number evaluated by quantitative PCR does not appear to correlate with the clinical outcome of patients, whereas the result of the analysis by fluorescence *in* *situ* hybridization (FISH) appears to be associated with an increase in treatment response [32]. However, the predictive value is uncertain, and further studies are required to assess the increase of EGFR gene copy number as a predictive biomarker of response to anti-EGFR therapy. Activating mutations in the EGFR catalytic domain plays an important role in determining the responsiveness to anti-EGFR therapy in lung cancer. However, mutations in the EGFR tyrosine kinase domain are considered to be extremely rare in patients with CRC [33] and they are not significantly associated with the clinical response of metastatic CRC to anti-EGFR mAb [24].

The overexpression of alternative EGFR ligands, such epiregulin and amphireguline, may promote tumor growth and survival by an autocrine loop [34]. Several studies have correlated the expression of these ligands with sensitivity to cetuximab monotherapy. The results showed a statistically longer progression-free survival (PFS) period among patients with high expression of epiregulin. The exclusive use of an amphiregulin or epiregulin gene expression profile does not, however, result in the selection of patient populations benefiting from cetuximab treatment [35].

Scartozzi *et al.,* investigated the correlation between the efficacy of irinotecan plus cetuximab therapy and methylation status in the *EGFR* promoter [36]. Patients with tumors harboring the hypermethylating *EGFR *promoter experienced a worse clinical outcome in terms of progression-free survival (PFS) and overall survival (OS), suggesting that the methylation of *EGFR *promoter plays a role in determining the efficacy of anti-EGFR mAb. Thus, the hypermethylation of *EGFR *promoter may be a valuable and important indicator that should be considered in further investigations of the role of EGFR as a therapeutic target in patients with CRC.

Collectively, the predictive value of alterations in EGFR expression level remains unconvincing in the use of anti-EGFR therapy. Therefore, the focus has shifted to alterations of the key signaling pathway downstream of EGFR.

## BIOMARKERS DOWNSTREAM OF EGFR

The constitutive activation of signaling pathways downstream of EGFR drive the growth and progression of CRC and provide an escape mechanism that allows tumors to overcome the pharmacological blockade induced by anti-EGFR mAb [37]. *KRAS*, *BRAF*, *PTEN*, and *PI3KCA* mutations have been highlighted as the mechanisms that activate EGFR signaling pathway.

*KRAS* is a proto-oncogene encoding a small 21-kD guanosine triphosphate (GTP)/guanosine diphosphate (GDP) binding protein involved in the regulation of the cellular response to many extracellular stimuli [38]. After binding and activation by GTP, *KRAS* recruits the oncogene *BRAF*, which phosphorylates MAP2K (mitogen-activated protein kinase kinase), thereby initiating MAPK signaling leading to the expression of the protein involved in cell proliferation, differentiation, and survival [39]. *KRAS* is the mostly commonly mutated gene in this pathway, and 35%–45% patients with CRCs carry this mutation, which is an early event in colon tumorigenesis [40]. *KRAS* mutations frequently induce glycine-to-valine substitutions at the catalytic sites of amino acids, which leads to the loss of GTPase activity and subsequent continuous binding of GTP to RAS. This constitutive activation of RAS results in the dysregulation of the downstream RAS-ERK signaling pathway independently of EGFR. Similarly, the kinase activity of the BRAF mutant protein is greatly elevated, which also constitutively stimulates downstream ERK activity independently of RAS and EGFR. Thus, the constitutive activation of *KRAS* or *BRAF* mutation leads to EGFR-independent tumorigenicity in patients with CRC. Therefore, the oncogenic activation of the RAS signaling pathway impairs the response of colorectal cancer cells to cetuximab [6-10, 41].

The PTEN/PI3K/Akt pathway also affects several cellular processes such as cell proliferation, apoptosis, and invasion [42]. Signal transduction through this pathway is mediated by conversion of phosphatidylinositol bisphosphate (PIP2) to phosphatidylinositol triphosphate (PIP3) by phosphatidylinositol 3 kinases (PI3K) following their activation, and this reaction is antagonized by phosphatase and a tensin homolog deleted on chromosome ten (PTEN). The *PTEN* mutation is known to correlate with microsatellite instability (MSI-H) in patients with CRC [43, 44]. Of the genes that encode the enzymatic subunit of PI3K heterodimers, the *PIK3CA* gene that encodes the p110 subunit of PI3K has been found to be most frequently activated by its mutations in some human cancers [42]; this promotes AKT1 phosphorylation to activate a parallel intracellular axis [45]. 

Several reports have suggested that there is cross-talk between Ras/Raf/MAP/MEK/ERK and/or PTEN/PI3K/Akt pathways. Specifically, PIK3CA can be activated *via *interaction with the RAS protein [46].

## SPECTRUM OF THE *KRAS* AND *BRAF* GENOTYPES IN PATIENTS WITH CRC 

Estimates of the *KRAS* mutation frequency in metastatic CRCs are based on selective clinical studies or drug admission trials with variable inclusion criteria. According to previous investigations on the spectrum of the *KRAS* genotype in our database of CRC cases, the most frequent mutations at the *KRAS* codon 12 were G12D, G12V, G12R, G12C, G12S, and G12A, which accounted for more than 95% of the codon 12 mutations. The G13D and G13C mutations at codon 13 and the G61H, G61L, G61E, and G61K mutations at codon 61 were the most common mutations that occurred at these codons [47]. All these *KRAS* mutations have been previously described as oncogenically active and they are present in the COSMIC (catalog of somatic mutations in cancer) database [48]. Data from a large Japanese population of patients with advanced and recurrent CRC revealed that *KRAS* mutations were present in approximately 35% patients with CRC of which 25% patients had mutations at codon 12 and 10% patients had mutations at codon 13. This observation was consistent with that of previous studies on selected cohorts that reported frequencies in the range of 30%–42% [47].

Although more than 40 somatic mutations have been described in the *BRAF* kinase domain, the most common mutation across various cancers is the classic GTG→GAG substitution at position 1799 of exon 15, which results in the V600E amino acid change and subsequent constitutive activation of the EGFR signaling pathway. Functionally, this is the most important mutation involved in the receptor-independent aberrant activation of the EGFR signaling pathway and CRC carcinogenesis. Recent studies in western countries suggested that *BRAF* mutations occur in 10%–20% of patients with sporadic diseases [8, 9, 10, 49, 50]. *BRAF* V600E mutation in the Japanese population was observed in 4.7% patients with CRC; this appeared to be lower than that found in western populations. None of the patients with CRC carried both *KRAS* and *BRAF* mutations, supporting the hypothesis that *KRAS* and *BRAF* mutations are mutually exclusive [51-53]. One possible explanation for the comparatively low frequency of *BRAF* mutations might be the difference in ethnicities. Indeed, several studies reported that the mutation rates of DNA mismatch repair (MMR) genes, such as *hMSH2* and *hMLH1*, in hereditary non-polyposis colorectal cancer (HNPCC) varies across countries. Therefore, geographical variation may account for the differences in the mutation spectrum of *BRAF*, as observed for MMR genes [54-56].

## *KRAS* TESTING IS A SCREEN FOR DRIVER MUTATION, BUT NOT FOR SUPER-RESPONDER

**Fig. (1):**
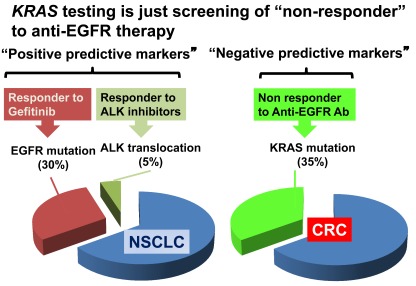
*KRAS* testing is a screening of non-responders to anti-EGFR therapy. Abbreviations: NSCLC, non-small cell lung cancer; CRC, colorectal cancer.

Routine *KRAS*/*BRAF* screening should be performed before initiating anti-EGFR therapy in patients with CRC to predict non-responsiveness to anti-EGFR therapy and to prevent drug-induced toxicity. In addition, limiting the use of anti-EGFR mAb to patients with wild-type, i.e., non-mutated, *KRAS* testing may result in avoiding heavy expenses [57]. Therefore, optimal *KRAS*/*BRAF* geno-typing procedures with pathological specimens are necessary. The significance of *KRAS*/*BRAF* mutations as predictive markers in patients with CRC should be considered while selecting a method for *KRAS* testing. Patients with non-small cell lung cancer (NSCLC) show 30% EGFR mutations and 5% ALK translocations, which are driver mutation targeted by molecular target agents such as Gefitinib or ALK inhibitors. Because the populations with these gene alterations are super responders to specific molecular target therapies, screening for driver mutations is essential and requires a technique with high sensitivity. In patients with CRC, *KRAS* testing is also a screen for a driver mutation. However, unlike NSCLC, the main purpose of *KRAS* genotyping in patients with CRC is screening for absolute non-responders to anti-EGFR mAb (Fig. **1**).

Allele-specific PCR methods such as TaqMan MGB Probes or ScorpionsARMS are available as commercial test kits, and some clinical trials have applied this method for *KRAS* genotyping. ScorpionsARMS is believed to be more sensitive than both direct sequencing and cycleave PCR and can detect mutations in samples containing 1% mutant allele sequences. However, optimal *KRAS* genotyping methods may not necessarily be highly sensitive. The most critical mistake that should be avoided is an overestimation of the population with *KRAS* mutations that would lead to depriving true responders of the benefits of anti-EGFR mAb. Furthermore, allele-specific PCR methods such as TaqMan MGB Probes or ScorpionsARMS are too expensive to be used as routine diagnostic methods for *KRAS* genotyping [47].

Taken together, appropriate methods should be selected for *KRAS* genotyping, taking into consideration *KRAS* mutations as negative predictive biomarkers.

## ASSOCIATION OF *BRAF*/*KRAS* MUTATIONS WITH CLINICOPATHOLOGICAL FEATURES

Several reports suggested that tumors harboring *BRAF* mutations have different clinical and histopathological features compared with tumors harboring *KRAS* mutations. *BRAF* mutations occur more frequently in right-sided tumors [58-61]. A study evaluating the correlation between *KRAS*/*BRAF* mutational status and clinicopathological features in advanced and recurrent CRC also found that in 60% patients with CRCs having *BRAF* mutations, the tumor metastasized to the peritoneum compared with approximately 15% patients with CRCs with other subtypes. Furthermore, 60% *BRAF* mutation-positive specimens belonged to poorly differentiated adenocarcinoma or mucinous carcinoma subtypes [61]. It was recently reported that mucinous histology indicates a poor response to oxaliplatin- and/or irinotecan-based chemotherapies and is correlated with poor OS [62]. Because *BRAF* mutations are more frequent in mucinous carcinomas than in non-mucinous carcinomas as demonstrated by the present study and a previous study [63], the poor prognosis associated with mucinous histology may be at least partially explained by the poor prognosis of patients with CRC having *BRAF* mutations. These specific clinicopathological features support the hypothesis that *BRAF* mutation-mediated carcinogenesis in patients with CRC is initiated by altered BRAF function as an early step in the serrated pathway [64] that leads to the activation of EGFR signaling. In contrast to *BRAF* mutations, no significant differences have been observed in clinicopathological parameters based on the *KRAS* genotype in many studies, probably due to the lack of differentiation between *KRAS12* mutations and *KRAS13* mutations. However, an analysis in which a population of patients with CRC was categorized into four subtypes—*KRAS* and *BRAF* (wild/wild), *KRAS12* mutations, *KRAS13* mutations, and *BRAF* mutations (V600E), suggested that *KRAS13* mutations were also associated with right-sided tumors [61]. This suggests the possibility that *KRAS13* may have a phenotype distinct from that of other *KRAS* genotypes.

## PROGNOSTIC ROLE OF *KRAS *MUTATIONS

The prognostic value of *KRAS* mutations in patients with CRC remains controversial. Although the prognostic role of *KRAS* mutations has been previously investigated, no definitive conclusions have been drawn [65]. This may be because of differences in terms of study size, patient selection, tumor sampling, use of archival versus fresh/frozen material, laboratory methods, and data analyses. Furthermore, such prognostic analyses are performed mostly in homogeneous groups of metastatic patients with CRC treated with a specific chemotherapy regimen with or without cetuximab [14, 66] (Table **1**).

A recent translational study by Roth *et al.,* suggested that the prognostic value for *KRAS* mutation status for PFS and OS was lacking in PETACC-3, EORTC 40993, and SAKK 60-00 trials of patients with stage II and III resected colon cancer [22]. However, it has been reported that stage III patients having *KRAS* mutations displayed significantly worse disease-free survival compared with those having wild-type *KRAS* [50]. Furthermore, an N0147 trial assessing the potential benefit from cetuximab treatment combined with FOLFOX in patients with resected stage III CRC showed that the three-year disease-free survival in patients with wild-type *KRAS* was significantly better than that in patients with *KRAS* mutants (72.3% versus 64.2%, HR = 0.7, p = 0.004) (Table **1**). These analyses suggest that *KRAS* mutations are independent prognostic factors [67]. A Medical Research Council (MRC) COIN trial assessed the effects of cetuximab combined with oxaliplatin and fluoropyrimidine chemotherapy as a first-line treatment of patients with advanced CRC. This trial found that the median OS was significantly shorter in patients with *KRAS*, *NRAS*, or *BRAF* mutations (n = 706, 13.6 months) compared with those with wild types for *KRAS*, *NRAS*, and *BRAF* (n = 581, 20.1 months), irrespective of the treatment [68].

More importantly, few studies have differentiated *KRAS* mutations at codon 12 from those at codon 13 with respect to clinicopathological features and survival. Recent findings have suggested that CRC with a *KRAS* mutation is not clinically homogeneous but heterogeneous population [69]. This hypothesis may be supported by the fact that NSCLCs harboring alterations in the EGFR gene are biologically and pharmacologically heterogeneous. Indeed, there are differences in the transforming potential and EGFR tyrosine kinase inhibitor (TKI) sensitivity associated with EGFR somatic mutations L858R and the deletion mutant Del (746–750) in NSCLCs [70].

Collaborative RASCAL II studies were conducted to investigate the prognostic role of *KRAS* mutations in CRC progression. To explore the effect of *KRAS* mutations at different stages of CRC, 3493 patients were recruited in this multivariate analysis. RASCAL studies showed that tumors carrying a substitution of glycine to valine at codon 12, which was found in 8.6% patients, had a statistically significant impact on worse PFS (*p* = 0.0004, HR = 1.3) and OS (*p* = 0.008, HR = 1.29) [40]. This clinical data was supported by the finding that *KRAS12* mutations confer a more aggressive transforming phenotype than *KRAS13* mutations through a significant increase in the activation of AKT and expression of bcl-2, and a significant decrease in the expression of RhoA [71]. However, multivariate analysis by Bazan *et al.,* revealed that *KRAS13* mutations, but not other mutations, were independently related to the risk of relapse or death in a consecutive series of 160 untreated patients (median of follow up period = 71 months) who underwent resective surgery for primary CRC [72]. Consistent with this study, Yokota *et al.,* examined 229 patients with advanced and recurrent CRC who were treated with systemic chemotherapy, and demonstrated that the OS for patients with *KRAS13* mutations was significantly worse than for those who had wild-type *KRAS* and wild-type *BRAF*, whereas *KRAS12* mutation did not affect patient OS [61]. Furthermore, *KRAS*/*BRAF* genotype was analyzed in a large subgroup of 845 patients with metastatic CRCs who received FOLFIRI and FOLFOX chemotherapy with or without cetuximab as the first-line treatment in the CRYSTAL and OPUS studies, respectively [66]. The results revealed that *KRAS13D* mutations are associated with poor prognosis. Therefore, the finding that stage III patients with *KRAS* mutations displayed significantly worse disease-free survival than those with wild-type *KRAS* [50, 64, 67], might be partially explained by the impact of either *KRAS12* or *KRAS13* mutations on prognosis.

Taken together, differences in *KRAS* mutations at codons 12 and 13 may result in different biological, biochemical, and functional consequences that could influence the prognosis of CRC [72]. Larger studies are required to confirm whether a specific *KRAS* mutation might lead to a clinically relevant prognostic effect in patients with CRC.

## ARE *KRAS* MUTATIONS NEGATIVE PREDICTIVE BIOMARKERS FOR ANTI-EGFR mAB?

Several retrospective analyses have revealed that patients with *KRAS* mutations receiving first and subsequent lines of treatment do not respond to cetuximab or panitumumab, and that they show no survival benefit from such treatments [11-13, 15, 73]. *KRAS* mutations have emerged as a major predictor of resistance to anti-EGFR mAb in the clinical setting. Therefore, patients with metastatic CRC with *KRAS* codon 12- or *KRAS* codon 13-mutated tumors are presently excluded from treatment with anti-EGFR mAb.

However, one patient with a mutated *KRAS *tumor (1.2%) had a response in the CO.17 trial comparing cetuximab monotherapy with best supportive care (BSC) in patients with chemotherapy-refractory metastatic CRC [11]. Furthermore, a recent retrospective analysis by De Roock *et al.,* examined 579 patients with chemotherapy-refractory CRC who received cetuximab treatment, and revealed that patients with p.G13D-mutated tumors showed a trend toward a higher response rate than other *KRAS*-mutated tumors (6.3% versus 1.6%, *p* = 0.15). Strikingly, patients with *KRAS* codon p.G13D mutations who received cetuximab experienced longer progression-free and overall survival compared with BSC alone. In contrast, patients with other *KRAS* mutations did not appear to benefit from cetuximab. The authors suggested that p.G13D-mutated tumors may have a worse prognosis, based on the finding that patients with *KRAS* p.G13D mutations who received BSC alone showed significantly shorter survival compared with those with other *KRAS* mutations in the CO.17 study [74]. 

Furthermore, the association of *KRAS *p.G13D mutation with clinical outcome was investigated in a pooled analysis of patients from the CRYSTAL and OPUS studies. The population consisted of 689 patients in each treatment arm, including 447 versus 398 with wild-type *KRAS*, 41 versus 42 with *KRAS* p.G13D, and 201 versus 249 with other *KRAS* mutations, for chemotherapy alone and cetuximab plus chemotherapy arms, respectively. A heterogeneous treatment effect was observed with significant treatment interaction with the *KRAS* mutation status for response (*p* < 0.0001), PFS (*p* < 0.0001), and OS (*p* = 0.0219). In particular, the response rate in patients with *KRAS* p.G13D treated with cetuximab plus chemotherapy was better than that in those treated with chemotherapy alone (40.5% versus 22.0%; 95% CI, 0.90 to 6.45, *p* = 0.0748). The hazard ratio for PFS among patients with *KRAS* p.G13D was 0.60 (95% CI, 0.32 to 1.12, *p* = 0.1037), while that for OS among patients with *KRAS* p.G13D was 0.80 (95% CI, 0.49 to 1.30) in favor of the cetuximab plus chemotherapy arm. Although treatment effects were not statistically significant, patients with *KRAS* p.G13D had a similar relative treatment effect compared with patients with wild-type *KRAS*.

Taken together, these data may suggest that *KRAS* p.G13D mutations are poor prognostic biomarker, and the use of cetuximab may affect prolonged survival in patients receiving first-line chemotherapy and those with chemotherapy-refractory metastatic colon cancer. The clinical benefit of anti-EGFR therapy in patients with *KRAS* mutations, which are rare, may be partially explained by the benefit in p.G13D-mutated group. Further prospectively generated clinical investigations are necessary to confirm these data, because whether the *KRAS* p.G13D mutation is an effective negative predictive biomarker remains controversial. 

## PROGNOSTIC ROLE OF *BRAF* MUTATIONS

A *BRAF* mutation (V600E) has been studied in recent years for a better understanding of its possible role in prognosis and predicting the response to anti-EGFR mAb.

While few studies investigated the impact of *KRAS12* and *KRAS13* mutations on CRC prognosis, a series of recent studies confirmed the potential adverse prognostic impact of *BRAF* mutations (Table **2**). Yokota *et al.,* identified *BRAF* V600E mutation as an independent prognostic factor for survival in a representative cohort of 229 patients with advanced and recurrent CRC. The presence of this *BRAF* mutation was associated with a significantly higher risk of dying from cancer-related causes, independently of other factors such as age, gender, PS, *KRAS* status, pathological finding, number of metastases, and metastatic sites [61]. This finding is consistent with those of other recent studies using patients with both stage II and III disease and patients across all disease stages [21, 22, 50]. For example, an analysis of stage II and stage III patients with CRC [22, 50] was consistent with the finding that 44% population had recurrent disease [61]. Furthermore, *BRAF* mutations were correlated with survival in a heterogeneous group of patients with CRC that included all disease stages [20] (Table **2**). Furthermore, *BRAF* mutations are prognostic biomarkers for OS, particularly in patients with microsatellite instability (MSI), both low (MSI-L) and stable (MSI-S) tumors. In the high (MSI-H) subpopulation, a prognostic value of *KRAS* and *BRAF* mutation status was not found for RFS and OS [22] (Table **4**). Whereas *BRAF* mutations had no prognostic value in the relapse-free survival of stage II-III CRC, *BRAF* mutation is a strong determinant of OS after relapse [22].

Furthermore, the prognostic value of *BRAF* was analyzed in patients with CRC treated with specific chemotherapy regimens in clinical trials that evaluated a combination of cetuximab with chemotherapy (Table **3**). The CAIRO-2 study investigated a large series of metastatic patients with CRC treated with chemotherapy and bevacizumab with or without cetuximab in a subgroup of 520 patients. This study revealed that patients with CRC having *BRAF* mutations show a worse outcome, both in terms of PFS and OS, irrespective of the addition of cetuximab to the treatment [14]. The pooled analysis of the abovementioned CRYSTAL and OPUS studies revealed that the outcome of patients with CRC having *BRAF* mutations is worse than that of patients with CRC having wild-type *BRAF*, independently of treatment with cetuximab [66]. These findings further support the hypothesis that *BRAF* mutations are negative prognostic biomarkers. The *BRAF* genotype might be an additional stratification factor for future clinical trials of advanced and recurrent CRC. 

## PREDICTIVE ROLE OF *BRAF* MUTATIONS

Di Nicolantonio *et al.,* retrospectively analyzed objective tumor responses and survival, and the mutational status of *KRAS* and *BRAF* in 113 patients with metastatic CRC treated with cetuximab or panitumumab [9]. None of the *BRAF*-mutated patients responded to the treatment, while none of the responders carried *BRAF* mutations. *BRAF*-mutated patients had significantly shorter progression-free survival and overall survival than wild-type patients. The effect of *BRAF* V600E mutation on cetuximab or panitumumab response was also assessed using cellular models of CRC. The introduction of *BRAF* V600E allele impaired the therapeutic effect of cetuximab or panitumumab. Similarly, Souglakos *et al.,* addressed the predictive value of *BRAF* in 100 patients treated with cetuximab, including 8 in the first line, 37 in the second, and 55 in the third or higher, always in combination with chemotherapy [10]. No patients with a *BRAF*-mutant tumor responded to cetuximab, whereas objective responses were observed in 17% patients with wild-type *BRAF*. Patients with *BRAF* mutation also had a shorter PFS, regardless of whether cetuximab was administered in the second or third or higher lines. The effects of *BRAF* status on the efficacy of cetuximab plus chemotherapy were retrospectively analyzed in patients with metastatic CRC having wild-type *KRAS* [16, 17], indicating that the presence of *BRAF* mutation was significantly correlated with lower response rate than wild-type *BRAF*, with a response rate of 8.3% (2/24) in carriers of *BRAF* mutations versus 38.0% in *BRAF* wild types [17]. These results suggest that wild-type *BRAF* is required for the response to anti-EGFR mAb in metastatic CRC. However, these studies lacked data on *BRAF*-mutated patients treated with chemotherapy alone, so they failed to directly compare the efficacy of adding cetuximab or panitumumab to chemotherapy with that of chemotherapy alone in a cohort of *BRAF*-mutated patients. Therefore, whether *BRAF* and *KRAS* mutations are negative predictive biomarkers for anti-EGFR mAb cannot be ascertained.

In the pooled analysis of CRYSTAL and OPUS, patients with *BRAF* mutations seemed to benefit from the addition of cetuximab, with an increase of OS and a doubling of PFS rates, although this was not statistically significant [66]. The addition of cetuximab to FOLFIRI or FOLFOX regimens showed a trend towards better survival compared with FOLFIRI or FOLFOX alone. This result raises the possibility that the use of cetuximab might be effective for disease control at least as the first-line chemotherapy for patients with wild-type *KRAS* and mutant *BRAF*. 

Taken together, the association of *BRAF* mutations with the efficacy of anti-EGFR therapy remains controversial, but its significant negative prognostic value has been established. Such discrepant results among studies might be partially explained by the differential significance of *BRAF* mutations as predictive biomarkers for anti-EGFR mAb in the first-line and second-line or higher line chemotherapy. The relatively low frequency of *BRAF* mutations in patients with CRC makes it relatively difficult to draw absolute conclusions, but the present observations should be confirmed by examining an increased number of patients with *BRAF* mutations.

## CONCLUSIONS

The activation of EGFR signaling, such as Ras/Raf/MAP/MEK/ERK and/or PTEN/PI3K/Akt pathways, plays an important role in tumorigenesis and the tumor progression of CRC. Two predominant EGFR inhibitors have been developed including monoclonal antibodies that target the extracellular domain of EGFR and small molecule TKIs that target the receptor catalytic domain of EGFR. Although both classes of agents show clear antitumor activity, only the anti-EGFR mAb has been approved for clinical use in the treatment of patients with metastatic CRC. Because the predictive value of alterations in EGFR expression level is unclear in the use of anti-EGFR mAb, the focus has shifted to alterations of key signaling pathways downstream of EGFR. In particular, *KRAS* and *BRAF* mutations have been highlighted as the activating mechanisms of the EGFR signaling pathway. Routine screening for *KRAS*/*BRAF* genotype is extremely important for identifying patients with shorter survival in response to systemic chemotherapy, regardless of the use of anti-EGFR mAb, and for predicting patients who would benefit from anti-EGFR mAb treatment, which is costly and potentially toxic. However, the significance of *KRAS*/*BRAF* mutations as prognostic and/or predictive biomarkers in patients with CRC should be considered while selecting a method for *KRAS* genotyping.

*KRAS* mutations were observed in approximately 35% patients with CRC, of which 25% patients had mutations at codon 12 and 10% patients had mutations at codon 13. The *KRAS* genotype is a useful predictive biomarker for patients with metastatic CRC that is treated with anti-EGFR mAb. Recent reports have raised the possibility that *KRAS13* may have a specific phenotype that is different from other *KRAS* genotypes. Therefore, differences in *KRAS* mutations at codons 12 and 13 may result in different biological, biochemical, and functional consequences and clinical features, which may also influence the prognosis of CRC. Indeed, several retrospective analyses have suggested that *KRAS* mutations at codon 13, particularly *KRAS* p.G13D, as well as *BRAF* mutations are prognostic factors. Furthermore, a recent major research finding is that patients with *KRAS* p.G13D, but not other mutations, may experience a survival benefit from treatment with cetuximab plus chemotherapy. These findings also support the hypothesis that patients with CRC having *KRAS* mutations constitute a heterogeneous population. Since the prognostic and/or predictive role of *KRAS13* mutations continues to remain controversial, further prospective clinical investigations are warranted.

Several reports have suggested that tumors harboring *BRAF* mutations have distinct clinicopathological features. Importantly, *BRAF* mutations are significant negative prognostic biomarkers in patients with recurrent CRC across all disease stages. Moreover, the prognostic value of *BRAF* mutations has been confirmed in patients with CRC treated with specific chemotherapy regimens in clinical trials evaluating a combination of cetuximab with chemotherapy. However, whether *BRAF* mutations are negative predictive biomarkers for anti-EGFR mAb has not been ascertained, because the controlled study, which directly compared the efficacy of adding anti-EGFR mAb to chemotherapy with that of chemotherapy alone, is lacking in a small population with *BRAF* mutations. The application of novel strategies targeting *BRAF* kinase is warranted for the treatment of patients with CRC with *BRAF* mutations to improve their poor survival.

The mechanism of how anti-EGFR mAb functions is now being revealed. However, clinical data suggest that the Ras/Raf/ERK pathway is insufficient for completely predicting the response to anti-EGFR mAbs. Therefore, other factors, such as PIK3CA/PTEN deregulation and/or the expression status of epiregulin or amphiregulin, should also be focused on as possible predictive biomarkers for anti-EGFR mAb.

## Figures and Tables

**Table 1.** *KRAS* Mutation and Prognosis

Trial	Population	Therapy	Overall Survival (month)		HR	P-value	Prognostic ?
			KRAS wt	KRAS mut			
CO.17	3rd line	BSC	4.8	4.6	1.01	0.97	No
CAIRO-2	1st line	CapeOX+BV	22.4	24.9		0.82	No
N0147	Stage III	FOLFOX+Cmab	*72.3 %	*64.2 %	0.7	0.004	Yes
COIN	1st line	FU+OX+/-Cmab	17.5	14.4		<0.0001	Yes
Fariña-Sarasqueta, A. *et al*.	Stage III	-	?	?		0.03	Yes
PETACC-3	Stage II/III	FU/LV+/-CPT-11			1.09	0.48	No
FOCUS	1st line sequential	FU+/-CPT-11/OX	?	?	1.24	0.008	Yes
Van Cutsem, E. *et al*.	3rd line	BSC	7.6	4.4		**N.S.	No
EPIC	2nd line	CPT-11	11.56	10.68		**N.S.	No

* 3 year Disease free survival

** statistically not significant

Abbreviations: BSC, best supportive care; Cape, capecitabine; OX, oxaliplatin; Cmab, cetuximab; mut, mutated.

**Table 2.** *BRAF* Mutation and Prognosis

	Population	HR (95% CI)	Reference	Prognostic ?
Ann Oncol. 2010; 21(12):2396-402	stage II / III	0.45 (0.25–0.8)	*Mutant*	Yes
Gut 2009; 58: 90-96	All stage	1.20 (0.79–1.80)	*Wild*	Yes
PETACC-3	stage II / III	1.19 (0.84-1.69)	*Wild*	Yes
Br J Cancer 2011;104:856-62	Recurrent and advanced	4.25 (2.08–8.67)	*Wild*	Yes

Abbreviations: CT, chemotherapy; CB, CapeOX/bevacizumab; CBC, CapeOX/bevacizumab plus cetuximab.

**Table 3.** *BRAF* Mutation and Prognosis in the Clinical Trials Evaluating Combination of Cetuximab with Chemotherapy

	Population	Therapy	BRAF wt	BRAF mut	P-value	Prognostic ?
ASCO2010, Abstract No. 3506	1st line CRYSTAL/OPUS	CT	21.1	9.9	-	Yes
		CT+Cmab	24.8	14.1	-	
N Engl J Med. 2009; 361: 98-99	1st line CAIRO-2	CB	24.6	15.0	0.002	Yes
		CBC	21.5	15.2	0.001	

Abbreviations: CT, chemotherapy; CB, CapeOX/bevacizumab; CBC, CapeOX/bevacizumab plus cetuximab.

**Table 4.** Are *KRAS/BRAF* Mutations Predictive and/or Prognostic?

	KRAS mut			BRAF mut	
	Codon 12 mutant	Codon 13 mutant	Codon 61 mutant	MSI	MSS
Predictive marker	*Negative predictive*	*Negative predictive?*	*Negative predictive?*	*Positive predictive?*	
Prognostic marker	*No*	Yes?	?	No	Yes

Abbreviations: MSI, microsatellite unstable; MSS, microsatellite stable.
